# A Mental Health Surveillance System for the General Population During the COVID-19 Pandemic: Protocol for a Multiwave Cross-sectional Survey Study

**DOI:** 10.2196/23748

**Published:** 2020-11-26

**Authors:** Nasser F BinDhim, Nora A Althumiri, Mada H Basyouni, Asem A Alageel, Suliman Alghnam, Ada M Al-Qunaibet, Rasha A Almubark, Shahla Aldhukair, Yasser Ad-Dab'bagh

**Affiliations:** 1 Sharik Association for Health Research Riyadh Saudi Arabia; 2 College of Medicine Alfaisal University Riyadh Saudi Arabia; 3 Saudi Food and Drug Authority Riyadh Saudi Arabia; 4 Ministry of Health Riyadh Saudi Arabia; 5 College of Medicine Al-Imam Mohammad Ibn Saud Islamic University Riyadh Saudi Arabia; 6 King Abdulah International Medical Research Center Riyadh Saudi Arabia; 7 The National Center for Disease Prevention and Control Riyadh Saudi Arabia; 8 Saudi Health Council Riyadh Saudi Arabia; 9 King Fahd Specialist Hospital Dammam Saudi Arabia

**Keywords:** mental health, depression, anxiety, screening, surveillance, COVID-19

## Abstract

**Background:**

The COVID-19 outbreak can potentially be categorized as a traumatic event. Public health surveillance is one of the cornerstones of public health practice, and it empowers decision makers to lead and manage public health crises and programs more effectively by providing timely and useful evidence.

**Objective:**

This paper presents the protocol for a study that aims to identify, track, and monitor trends in the population in Saudi Arabia at risk of major depressive disorders and anxiety during the COVID-19 pandemic.

**Methods:**

This study utilizes continuous, cross-sectional, national-level mental health screening via computer-assisted phone interviews, conducted in four waves on a monthly basis (between May and August 2020). Arabic-speaking adults, aged ≥18 years, and living in Saudi Arabia were recruited via a random phone list. This surveillance system used the proportional quota sampling technique to achieve an equal distribution of participants, stratified by age and gender, and region, within and across the 13 administrative regions of Saudi Arabia. A sample size of 4056 participants per wave was calculated to achieve enough power to detect changes in mental health status. The questionnaire includes the Arabic version of the Patient Health Questionnaire-9 (PHQ-9) to measure depressive symptoms and the General Anxiety Disorder-7 (GAD-7) to measure anxiety. In addition, it will collect data on sociodemographic variables and potential risk factors.

**Results:**

Study recruitment began in May 2020. The data analysis was completed in October 2020, and the final report is expected to be published by the end of December 2020.

**Conclusions:**

Monitoring the population’s mental health status during the COVID-19 pandemic will inform decision makers of any potential deterioration in mental health on a national level and among subgroups, including across regions, age groups, and gender groups. It will allow decision makers to recognize issues and intervene sooner. It will also provide valuable scientific data to help understand the effects of epidemics and pandemics on mental health. As far as we know, this is the only study that attempts to monitor the mental health status of the general population on a monthly basis.

**International Registered Report Identifier (IRRID):**

DERR1-10.2196/23748

## Introduction

In January 2020, the World Health Organization (WHO) announced the outbreak of a new coronavirus disease—COVID-19 [1]. In March 2020, the WHO declared COVID-19 to be a pandemic, and this time of crisis began to generate mental stress in the population [1]. The COVID-19 outbreak can potentially be categorized as a traumatic event, as individuals in the community, in addition to isolation, can experience, witness, or be confronted with events that threaten death and/or serious injury to oneself or others [2-4]. The WHO’s Department of Mental Health and Substance Use issued a series of messages to support mental and psychosocial well-being in different target groups during the outbreak [1]. In addition, calls for immediate prioritization and collection of high-quality data on the mental health effects of the COVID-19 pandemic across populations and vulnerable groups were issued by mental health experts [3-6].

Although only a few studies have been published looking at the effect of the COVID-19 pandemic on the general population’s mental health on a national level, evidence of mental health deterioration is emerging. For example, in the United Kingdom, a national study covering 17,452 participants found that clinically significant levels of mental distress rose from 18.9% in 2018 to 27.3% in April 2020 [7]. Furthermore, a study in the United States found that in April 2020, 13.6% of adults reported symptoms of serious psychological distress, relative to 3.9% in 2018 [8]. These results suggest that the effect of COVID-19 on the mental health of the general population is significant. In addition, this effect has escalated within a short time in both of these countries, which demands further and more frequent investigations on trends of increase in psychological distress using standardized measurements.

Public health surveillance is one of the cornerstones of public health practice, and it empowers decision makers to lead and manage public health programs more effectively by providing timely and useful evidence [9]. Public health surveillance is defined as the systematic, ongoing collection, management, analysis, and interpretation of data, followed by timely dissemination of these data to public health programs to stimulate public health action [10]. Public health surveillance data can be utilized in many activities that are critical to public health research and practice. These activities include estimating the scope and magnitude of health problems, facilitating public health planning, identifying changes in health practices, detection of epidemics or public health crises, monitoring changes, and describing the natural history of a health event in a community [9].

Routine surveillance systems for mental health in many countries in the eastern Mediterranean region are rudimentary or absent, which makes it difficult to understand the needs of local populations and to plan accordingly [11]. Key components of mental health surveillance and information systems are (1) a national-level commitment to ensure that relevant high-quality information is collected and reported; (2) a minimum data set of key mental health indicators; (3) routine data collection that is supplemented by periodic surveys; (4) quality control; and (5) technology and skills to support data collection, sharing, and dissemination [11]. Mental health surveillance systems can be utilized in various situations by different professional groups, including public health and mental health practitioners, academic organizations, and decision makers. For example, officials have used mental illness surveillance data to track trends in mental illness and psychological distress associated with exposure to military combat or large-scale disasters [2,12]. Thus, surveillance data are imperative to the public health goals of reducing the incidence, prevalence, severity, and economic impact of mental health conditions via the provision of timely data to decision makers and the creation of opportunities for early intervention. Mental health screenings are now included in established health surveillance surveys, such as the Centers for Disease Control and Prevention’s (CDC) National Health Interview Survey (NHIS), the National Health and Nutrition Examination Survey (NHANES), and the Behavioral Risk Factor Surveillance System (BRFSS) [13].

National-level screening can be used to accurately estimate the prevalence of certain mental illness symptoms across populations, and by repeating surveys over time in a surveillance system, such screening can be used to detect and characterize mental health trends [12]. Screening generally cannot be used to diagnose mental health conditions with the same level of specificity as an individual clinical examination conducted by a psychiatrist [12]. Instead, data can be collected on a variety of subjective manifestations in changes in thinking, mood, behavior, and associated distress that correspond with clinical disorders [12]. Questionnaires that have been validated empirically to distinguish between persons with and without specific mental illnesses or general psychological distress are used in screening surveys to generate high-quality mental health data [12].

This paper presents the protocol for a study that aims to identify, track, and monitor trends on the population in Saudi Arabia at risk of major depressive disorders and anxiety during the COVID-19 pandemic. We also aim to assist decision makers to allocate resources and support where they are most needed.

## Methods

### Study Design

This study utilizes continuous, cross-sectional, national-level mental health screening via computer-assisted phone interviews, conducted in four waves on a monthly basis (between May and August 2020). This study used the QPlatform data collection system, which had integrated eligibility and sampling modules, to control the distribution of the sample [14]. All questions had to be answered for the questionnaire to be successfully submitted to the database. To ensure the quality of our mental health surveillance system, we considered all relevant attributes by evaluating the public health surveillance systems issued by the CDC [15]. The interview takes approximately 8 minutes to complete.

### Participants, Setting, and Recruitment

Participants were Arabic-speaking adults, aged ≥18 years, from Saudi Arabia. They will be recruited via a random phone number list generated from the Sharik Association for Health Research, a research participants’ database [16]. The Sharik database is a database of individuals interested in participating in health research that currently has more than 64,000 potential participants and grows on a daily basis, covering the 13 administrative regions of Saudi Arabia [16].

Participants were contacted by phone on up to three occasions. If the participant did not respond, another potential participant with a similar demographic profile (age, gender, region) was invited to participate. All data collectors received training on research ethics, data quality, and use of the data collection electronic system, as well as specific training on how to administer the screening tools used in this study.

### Sample Size

This surveillance system used the proportional quota sampling technique to achieve an equal distribution of participants, stratified by age and gender, and region within and across the 13 administrative regions of Saudi Arabia. We used two age groups based on Saudi Arabia’s median adult age of 36 years. This led to a quota of 52 strata for this study, which may have helped increase the diversity of the sample and reduced the risk of nonprobability sampling bias.

The sample size was calculated based on the depth of the subanalysis we needed to reach, which compares the age and gender groups across regions with a medium effect size of approximately 0.3 with 80% power and 95% CI [17]. Thus, each quota required 78 participants, and a total sample of 312 per region, to form a grand total of 4056 participants per wave. Once the quota sample was achieved, participants with similar characteristics were not eligible to participate in the study and were excluded automatically via the data collection system. Quota sampling is an automated process with no human interference, as the sampling process is controlled automatically by the data collection system, which eliminates the potential for human sampling bias [14].

### Questionnaire Design and Validation

Data collection included general demographic variables, such as age, gender, region, educational level, and marital status. It also included variables related to COVID-19, such as employment category (eg, health care professional, security, etc), concerns and worries about COVID-19, and COVID-19 incidence in family, friends, etc. In addition, other health-related risk factors, such as a history of noncommunicable diseases, obesity, physical activity, and smoking, were collected.

The main mental health screening tool used was the Patient Health Questionnaire-9 (PHQ-9) [18-20]. The PHQ-9 was selected over other depression screening tools since (1) it has been validated for use among various age groups, including adolescents, adults, and the elderly [21,22]; (2) it has been shown to have consistent performance regardless of the mode of administration (eg, patient self-report, interviewer-administered in person or by telephone, or touch-screen devices) [21,22]; (3) it has demonstrated validity and reliability when screening for depression, anxiety, and somatic and panic disorders in a Saudi sample [20,23,24]; and (4) it has been used for mental health screening in various international surveys and surveillance systems (eg, the US CDC uses the PHQ-9 in the BRFSS and the NHANES), which also allows for international comparison [12].

Finally, anxiety was measured using the Generalized Anxiety Disorder-7 (GAD-7), which has also shown good validity and reliability in various studies [25]. The GAD-7 also demonstrated good validity for general population screening, including in the Arabic language among the Saudi population [26-29].

After the first draft of the survey was finalized, a linguistic validation was conducted via a focus group of 8 participants, who were asked to discuss and answer the survey as a group. According to the results of the focus group and feedback from the researchers and interviewers, the questionnaire was further edited, and a final version was produced. Following this, a pilot stage study with a small sample size will be conducted via phone interview to assess internal consistency and test the surveillance system operation plan.

### Outcome Measures

To determine the prevalence of the high risk of depression and anxiety in our sample, we used two PHQ-9 thresholds recommended in the literature. The first threshold is a score >10, comprising pooled estimates of 10 studies with the best trade-off between sensitivity (0.89, 95% CI 0.75-0.96) and specificity (0.89, 95% CI 0.79-0.94) [30]. The second threshold is a score ≥15, which has shown the highest specificity at 0.96 (95% CI 0.94 to 0.97) [30].

In terms of the GAD-7, pooled sensitivity and specificity values appeared acceptable at a cutoff point of 8 (sensitivity: 0.83, 95% CI 0.71-0.91; specificity: 0.84, 95% CI 0.70-0.92), and cutoff scores between 7 to 10 also had similar pooled estimates of sensitivity and specificity [27]. In addition, on the GAD-7 anxiety scale, a score of ≥10 was deemed to be the optimum cutoff in the literature and according to previous studies of the Saudi population [25,29].

### Statistical Analysis

Data were weighted to equal the adult population in Saudi Arabia, according to the General Authority of Statistics 2017 Census Report [31]. Quantitative variables will be presented by mean and SD values if they have a normal distribution, or median and range, as appropriate, and will be compared using the *t* test. Categorical variables will be presented as percentages and CIs, and will be compared using the Pearson chi-square test. As this study uses automated electronic data collection, there are no missing values; the QPlatform also includes a data integrity check to prevent users from entering invalid data (eg, the maximum age is 99) [14].

### Ethical Considerations

The study was performed in accordance with the Declaration of Helsinki and followed the Saudi Arabia Research Ethics Standards. Consent was obtained verbally at the beginning of the phone interview. All of the obtained data are deidentified and cannot be used to identify individual participants. Ethics approval was obtained from the Sharik Association for Health Research institutional review board (approval number: 01-2020).

## Results

### Project Timeline

The first wave of the project started in the third week of May 2020. The next waves began in the third week of each month (June, July, and August). Data collection took around 2 weeks for each wave. The data analysis was completed in October 2020, and the final report is expected to be published by the end of December 2020. The results will be reported according to the STROBE (Strengthening the Reporting of Observational Studies in Epidemiology) checklist for cross-sectional studies [32].

### Dissemination

The main study outcome will be disseminated to decision makers via a statistical dashboard developed for this project. The dashboard was updated within 72 hours after completion of data collection each month to ensure that the findings reached decision makers in a timely manner. The findings from this study will be disseminated locally and internationally through publication in peer-reviewed journals and conference presentations at the national and international levels.

### Data Availability

Once the project is completed, data will be available upon request via the Saudi National Health Research Center.

## Discussion

### Overview

The COVID-19 pandemic has created a complicated system of stressors affecting the general population in a multilayered manner, including the following: abrupt changes to lifestyle, uncertainties about the future, deterioration of livelihood, social isolation, imposed quarantine, stigmatization, loss of loved ones, deprivation of culturally appropriate mourning rituals, and, finally, the threat of contracting COVID-19 [4]. In addition, generalized fear and fear-induced overreactive behaviors among the public may impede infection control. Thus, monitoring mental health status during the COVID-19 pandemic can inform decision makers about any potential deterioration in mental health on a national level and among subgroups, including across regions, age groups, and gender groups [33]. It will allow decision makers to recognize issues and intervene sooner, as well as identify vulnerable populations to provide tailored mental health interventions to potentially produce better outcomes. This project is among the first large-scale mental health surveillance system in Saudi Arabia. The continuous monitoring of mental health status during the COVID-19 pandemic will assist in understanding the effect of various social and economic measures implemented to control disease transmission, as will the effects of mental health interventions. In addition to its main aim, it will provide specific, valuable lessons to learn from for the future development of surveillance systems for mental health and for other public health issues.

Upon the success of this surveillance system, it may be linked to electronic pathways to provide clinical diagnosis and telemedicine-based interventions to treat and manage clinical and subclinical mental health issues. Telemedicine-based programs can play an important role during the COVID-19 crisis, given their unique potential for scalability [34]. There is already established evidence supporting the general population’s willingness to use a mental health app and discuss their results with their doctors, which can then lead to a clinical diagnosis of mental health conditions [21,22].

As COVID-19 may develop into a cultural or societal trauma, the monitoring of mental health status in the general population plays an important role in postpandemic recovery efforts [4]. For example, knowing when there is an increase in mental health conditions and the types of these conditions in society can help in the planning and scaling of mental health services, and stimulate innovation in mental health intervention delivery. This can help increase the chances of successful recovery efforts and transition to normal life after COVID-19. This can also provide an opportunity and prepare society to deal effectively with future population-level crises. At the same time, it is a chance to increase the population’s awareness of the importance of mental health and related issues, like mental health stigmatization.

The lack of a national screening baseline in Saudi Arabia generally, and, specifically, using the same screening tools as in this project, poses a challenge to understanding the effect of the first wave of COVID-19; however, the authors assume that, with the continuous health and economic measures implemented during the study period and given the sensitivity of the screening tools to mental health changes, the accumulation of data from the four waves will provide a clear picture of the mental health effects of COVID-19.

The use of proportional quota sampling provides more statistical power to detect changes, not only at national levels but also at regional levels, which can help further in stratifying data in relation to the most affected regions and subpopulations in order to provide a more in-depth picture of the effects of COVID-19. However, we acknowledge that using nonprobability sampling involves some risk of bias. Currently, in Saudi Arabia, the only way to conduct a random representative national survey is via household interviews. However, such a method is not possible during COVID-19 restrictions and lockdowns; it is also very expensive to implement this on a monthly basis. Thus, this study also considered the cost of conducting such a project, which becomes more cost effective via quota sampling. Finally, to improve the sampling accuracy, 52 strata were used to allow for the inclusion of a more diverse sample.

### Conclusions

This mental health surveillance system protocol provides a comprehensive approach to monitor the mental health status of the general population on a monthly basis during the COVID-19 pandemic. It was developed utilizing the highest standard of public health surveillance systems. The results of this project will provide valuable data for decision makers about mental health status changes during different phases of the epidemic in Saudi Arabia. It will also provide valuable scientific data to help in understanding the effect of such crises on mental health, since this is, to the best of our knowledge, the only study that attempts to monitor the mental health status of the general population on a monthly basis.

## Abbreviations

BRFSS: Behavioral Risk Factor Surveillance System
CDC: Centers for Disease Control and Prevention
GAD-7: General Anxiety Disorder-7
NHANES: National Health and Nutrition Examination Survey
NHIS: National Health Interview Survey
PHQ-9: Patient Health Questionnaire-9
STROBE: Strengthening the Reporting of Observational Studies in Epidemiology
WHO: World Health Organization
